# Combined association of birth weight and current overweight/obesity with cardiometabolic risk factor clustering in Chinese children and adolescents: a cross-sectional study

**DOI:** 10.3389/fendo.2026.1917806

**Published:** 2026-09-09

**Authors:** Gang Tian, Ruiheng Zhang, Jialin Sun, Xuejiao Chen, Xiaozhuan Liu, Yajing Guo, Mengwei Wang, Li Gao, Chen Zhang, Huiyu Du, Zhicheng Han, Jialiang Zhu, Dandan Tian, Jingge Zhao, Linqi Diao, Min Liu, Yibin Hao

**Affiliations:** 1 Henan Province Hypertension Precision Prevention and Control Engineering Research Center, Henan Provincial People’s Hospital, Zhengzhou University People’s Hospital, Zhengzhou, Henan, China; 2 College of Public Health, Zhengzhou University, Zhengzhou, Henan, China; 3 Department of Hypertension, Henan Provincial People’s Hospital, Zhengzhou University People’s Hospital, Zhengzhou, China; 4 Henan Provincial Center for Disease Control and Prevention, Zhengzhou, China; 5 Henan Engineering Technology Research Center for Prevention and Treatment of Panvascular Diseases, Henan Provincial People’s Hospital, Zhengzhou University People’s Hospital, Zhengzhou, Henan, China; 6 Department of Cardiology, Henan Provincial People’s Hospital, Zhengzhou University People’s Hospital, Zhengzhou, Henan, China; 7 Department of Clinical Research Center, Henan Provincial People’s Hospital, Zhengzhou University People’s Hospital, Zhengzhou, Henan, China

**Keywords:** birth weight, cardiometabolic risk factor clustering, childhood obesity, cross-sectional study, interaction

## Abstract

**Introduction:**

While early growth and current weight individually affect cardiometabolic health, their combined effects are poorly understood. This study examines the integrated impact of birth weight and current weight status on the clustering of cardiometabolic risk factors (CMRFs) in children and adolescents.

**Methods:**

This study analyzed data from 15,910 children and adolescents aged 6 to 18 years from a multistage cross-sectional survey. Participants were categorized into six combined exposure groups based on birth weight (normal/low/high) and current weight status (non-overweight/overweight/obese). Multivariable logistic regression models assessed the associations of these categories with CMRF clustering (defined as the presence of 2 or more of the following: elevated blood pressure, impaired fasting glucose, elevated triglycerides, and low high-density lipoprotein cholesterol). Additive interaction was evaluated using the relative excess risk due to interaction (RERI), attributable proportion (AP), and synergy index (SI). Statistical mediation analysis decomposed the association of birth weight with CMRF clustering into indirect (current BMI) and direct pathways.

**Results:**

Of 15,910 participants, 6.30% had CMRF clustering. In fully adjusted models, compared with the normal birth weight/non-overweight group, the low birth weight/overweight-obese group had the highest odds of CMRF clustering (OR = 6.11, 95% CI: 3.32 to 10.53, *P* < 0.001). No statistically significant additive interaction was observed between low birth weight and current overweight/obesity (RERI = 1.57, *P* = 0.193; AP = 0.26, *P* = 0.127; SI = 1.44, *P* = 0.158). Mediation analysis revealed a competitive mediation pattern: birth weight had a significant positive indirect effect on CMRF clustering through current BMI (*β* = 0.0091, *P* < 0.001) and a significant negative direct effect (*β* = −0.0144, *P* = 0.002), while the total effect was not statistically significant (*β* = −0.0052, *P* = 0.326).

**Conclusion:**

Although the combination of LBW and current overweight/obesity conferred the greatest risk for CMRF clustering, this was driven primarily by current weight status rather than a synergistic effect. However, LBW remained independently associated with metabolic risk after BMI adjustment, indicating distinct pathophysiological pathways. Thus, preventing excessive weight gain in low birth weight children is critical for reducing cardiometabolic risk.

## Introduction

The global prevalence of overweight and obesity among children and adolescents has risen substantially over the past three decades. Pooled estimates indicate that, in 2022, over 390 million children and adolescents aged 5–19 were living with overweight or obesity (1). This trend is particularly pronounced in China. Data show that, in 2020, nearly 20% of children and adolescents aged 6 to 17 were overweight or obese, with an obesity rate of approximately 7.9% (2). Childhood obesity is strongly associated with cardiometabolic risk factors (CMRFs), including elevated blood pressure, dyslipidemia, and impaired glucose metabolism (3, 4). Importantly, the co-occurrence (clustering) of multiple CMRFs confers substantially greater cardiovascular risk than any single factor alone, and evidence indicates that such clustering in childhood tracks into adulthood, predicting premature cardiovascular events (4, 5). Understanding the determinants of CMRF clustering in pediatric populations is therefore of critical public health importance. The precise identification of high-risk metabolic phenotypes in early life and the elucidation of their evolutionary pathways have thus become urgent public health priorities. The Developmental Origins of Health and Disease (DOHaD) hypothesis, originally proposed by Barker et al. (6), posits that adverse intrauterine conditions – reflected by indicators such as low birth weight (LBW)–program permanent structural and metabolic adaptations that increase susceptibility to cardiometabolic diseases in later life. Subsequent refinements of this framework, including the “thrifty phenotype” hypothesis (7) and the “predictive adaptive response” model (8), have further proposed that when early nutritional deprivation is followed by postnatal nutritional abundance, a mismatch occurs that may amplify metabolic risk through mechanisms such as preferential central fat deposition, insulin resistance, and reduced nephron endowment (9, 10). Conversely, high birth weight (HBW), which is often indicative of intrauterine overnutrition or gestational diabetes, has also been linked to an increased risk of childhood obesity. However, the independent association of HBW with CMRFs remains less consistent (11, 12).

Despite the growing body of evidence linking birth weight and childhood obesity separately to cardiometabolic outcomes, few studies have systematically examined their joint effects on CMRF clustering. Among the limited available studies, most have focused on adult populations and may not be directly generalizable to pediatric settings, given the distinct metabolic and developmental characteristics of children (13). Several investigations that did include younger populations used birth weight as a continuous variable without examining categorical joint exposures, potentially obscuring threshold effects and clinically meaningful risk stratification (14). Moreover, few studies have formally tested for biological interaction on the additive scale, which is considered the more appropriate measure for evaluating public health significance of joint exposures. Furthermore, whether the elevated cardiometabolic risk observed in children with a combination of low birth weight and current overweight/obesity reflects a synergistic (supra-additive) interaction or merely the additive accumulation of two independent risk factors remains an unresolved question (15).

Therefore, leveraging data from a large-scale cross-sectional survey of children and adolescents in Henan Province, China, this study aimed to quantify the joint associations of birth weight categories (low, normal, and high) and current weight status (non-overweight versus overweight/obese) with CMRF clustering, and to further assess whether low birth weight and current overweight/obesity exhibit additive interaction on CMRF clustering risk. Beyond evaluating joint and interactive effects, this study also sought to decompose the total statistical association of birth weight with CMRF clustering into a direct pathway independent of current adiposity and an indirect pathway operating through current BMI as a potential statistical mediator.

## Methods

### Study design and participants

**Figure 1:**
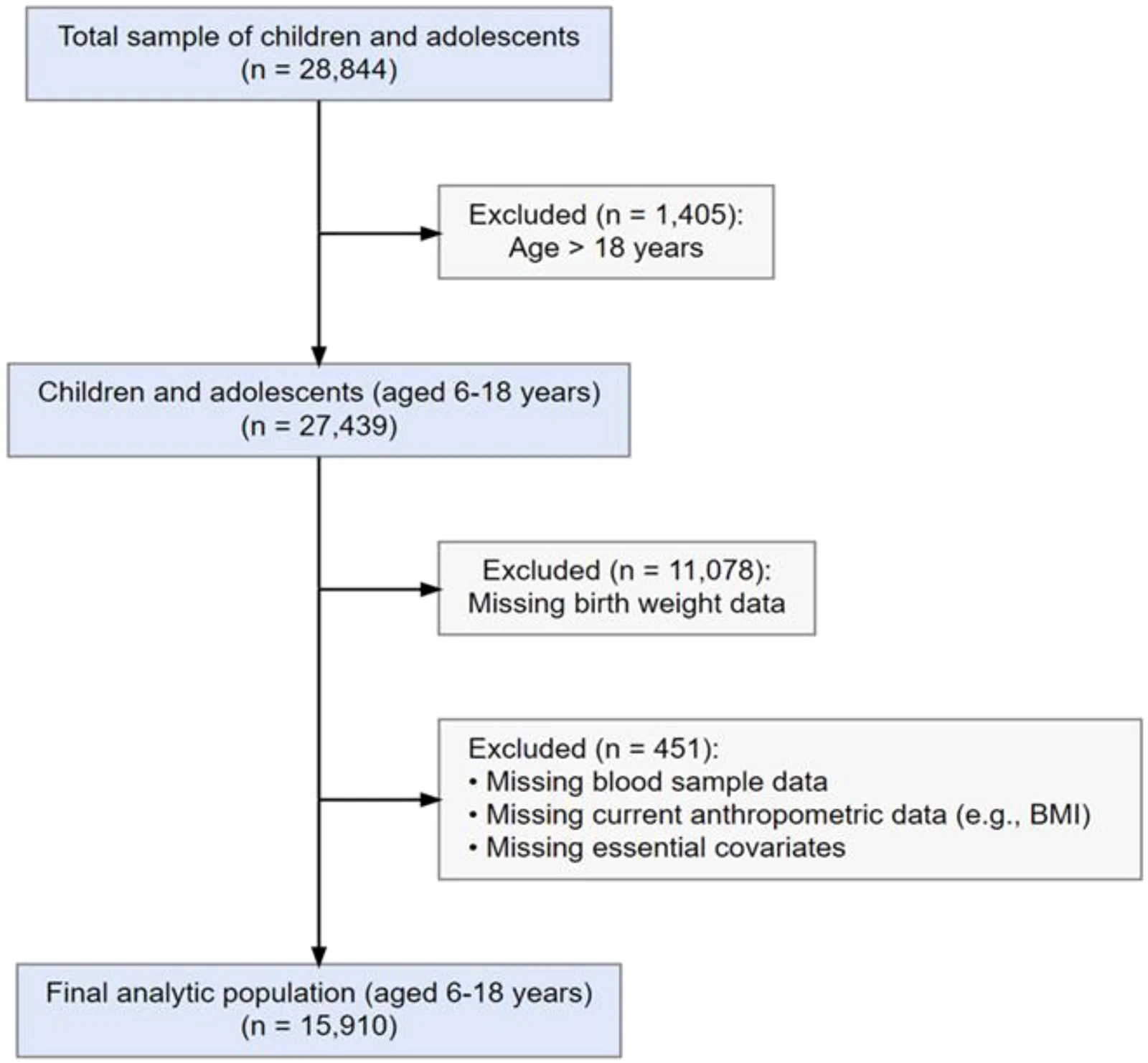
Selection of study population.

The present study is predicated on the Epidemiological Survey of Chronic Diseases among Children and Adolescents in Henan Province, a multistage stratified cluster cross-sectional survey conducted in central China in 2023, covering 18 prefecture-level cities (including Jiyuan, a province-administered county-level city). The objective of this study is to elucidate the prevalence and distribution patterns of cardiometabolic risk factors among children in Henan Province, as well as their potential influencing factors. This multistage cross-sectional survey enrolled 28,844 children and adolescents, of whom 15,910 aged 6 to 18 years were retained for the final analysis. A multistage stratified cluster random sampling approach was used. In the first stage, the seventh national population census data of Henan Province were used to construct the first-stage sampling frame, and one district/county was selected from each of the 18 prefecture-level cities by probability proportional to size (PPS) sampling, yielding 18 survey sites. In the second stage, an education-institution sampling frame was constructed from all primary, junior, and senior high schools in each survey site, and two primary schools, two junior high schools, and two senior high schools were selected per site by simple random sampling (108 schools in total). In the third stage, 1–2 classes per grade were randomly selected by cluster sampling through the survey information platform, and, provided that at least 65 students per grade were ensured, all students in the selected classes were invited to participate (excluding students with cognitive, mental, or hearing impairments) in questionnaire surveys, physical examinations, and blood sample collection. Based on the sample size calculation (allowing for a 10% non-response rate), the target total sample size was approximately 26,000, with at least 1,500 participants per survey site. Regarding participation, the survey protocol specified that the individual-level refusal rate should be controlled within 10%; because whole classes served as the basic sampling units, response rates were recorded at each site by the local Center for Disease Control and Prevention according to the protocol but were not aggregated or reported at the analysis level. A total of 28,844 children and adolescents participated in this study. However, cases were excluded if they were older than 18 years (n=1,405), lacked birth weight data (n=11,078), or had missing core anthropometric measurements or blood indicators (n=451). The final analysis included 15,910 participants aged 6 to 18 years (**Figure 1**). As the sampling frame consisted of primary, junior, and senior high school students (who generally enter primary school at approximately 6 years of age in China), no participants younger than 6 years were included in the sampling frame. The ethical approval of this study was undertaken by the Medical Ethics Committee of Henan Provincial Center for Disease Control and Prevention, with the approval number 2022-KY-006-02. Written informed consent was obtained from the parent/guardian of each participant. As this was an observational cross-sectional survey without any intervention, no clinical trial registration was required.

### Questionnaire survey

This study collected information through student and parent questionnaires. The student questionnaire covered basic student information, including moderate-to-vigorous physical activity, screen time, sleep duration, carbonated beverage consumption, and smoking and drinking habits. The parent questionnaire included basic household information, student birth weight, and the educational background of the student’s primary caregiver. Students completed the questionnaire on-site using tablets under the guidance of trained investigators, while parents completed theirs online by scanning a dedicated QR code. The questionnaires incorporated built-in logic jumps and error-checking programs to maximize data completeness and validity. All questionnaires were uniformly designed by the provincial project working group and underwent logical testing and expert review prior to formal implementation. This ensured scientifically sound content, structurally complete formats, and clear language tailored to the cognitive characteristics of children and adolescents, thereby safeguarding the feasibility of the survey and the validity of the data.

### Anthropometric measurement and blood sample detection

All measurements were performed by trained investigators following standardized physical examination protocols. Subjects removed shoes and hats as instructed and wore single-layer lightweight clothing. Height and weight were measured using a TZG-type stadiometer (accurate to 0.1 cm) and an electronic scale (accurate to 0.1 kg), respectively. Waist circumference was measured twice at the end of a relaxed exhalation using a soft tape measure (accurate to 0.1 cm), with the average recorded. Blood pressure was measured using a clinically validated Omron electronic sphygmomanometer. After 5 minutes of seated rest, three consecutive measurements were taken (1-minute intervals between each), with the average incorporated into the analysis.

After fasting for at least 8 hours, fasting venous blood samples were centrally collected by professional medical personnel. Blood samples underwent standardized static separation and centrifugation before being stored in a dedicated cold chain at 2–8 °C. Testing was conducted centrally on the same day by a nationally accredited third-party medical laboratory. Core metabolic risk assessment indicators included fasting blood glucose (FBG), triglycerides (TG), and high-density lipoprotein cholesterol (HDL-C).

Strict quality control measures were implemented throughout the survey. All measuring instruments complied with national metrology certification requirements, and operators underwent standardized training and passed qualification assessments. Daily random retesting was conducted on 5% of subjects. When results failed to meet standards, that day’s data was invalidated and remeasured to ensure accuracy and reliability.

### Birth weight

Birth weight was reported by the primary caregiver via a standardized questionnaire administered during the survey. Caregivers were instructed to refer to the child’s birth medical certificate when available. Birth weight was categorized into three groups: low birth weight (LBW, <2,500 g); normal birth weight (NBW, 2,500–3,999 g); and high birth weight (HBW, ≥4,000 g) (12).

### Current weight status

Body mass index (BMI) was calculated as weight in kilograms divided by the square of height in meters (kg/m^2^). According to the Chinese national reference standards for screening overweight and obesity in school-aged children and adolescents (WS/T 586-2018) (16), children and adolescents were classified as underweight, normal weight, overweight, or obesity based on age- and sex-specific BMI cutoff values. For the primary analyses, current weight status was dichotomized into “non-overweight” (including underweight and normal weight) versus “overweight/obesity” (including overweight and obesity), consistent with the reference category used in the joint-exposure analyses. We combined underweight and normal weight into a single reference category because this study focuses on comparing non-overweight versus overweight/obese weight states, and because underweight participants constituted only a very small proportion (2.45%) of the study sample.

### Clustered CMRFs

CMRF clustering was defined as the simultaneous presence of two or more of the following four components: (1) Elevated blood pressure: systolic blood pressure (SBP) and/or diastolic blood pressure (DBP) ≥ 95th percentile for age, sex, and height, according to the Chinese national reference (17); (2) Impaired fasting glucose: fasting blood glucose (FBG) ≥ 5.6 mmol/L, according to the American Diabetes Association criteria adopted for Chinese children (18); (3) Elevated triglycerides: triglycerides (TG) ≥ 1.70 mmol/L (19); (4) Low high-density lipoprotein cholesterol: HDL-C ≤ 1.04 mmol/L (19). Notably, obesity-related anthropometric indicators (e.g., waist circumference, BMI) were intentionally excluded from the CMRF clustering definition to avoid definitional overlap with the exposure variable (current overweight/obesity status), which could artificially inflate the observed associations. This approach ensures that the outcome captures metabolic derangements independent of adiposity per se.

### Covariates

Sociodemographic and lifestyle variables, including residence, sex, ethnicity, age, primary caregiver’s education level, boarding at school, moderate-to-vigorous physical activity, screen time, sleep duration, carbonated beverage consumption, smoking status, and alcohol intake, were considered as confounding variables. Primary caregiver’s education level was categorized as elementary school or below, high school, and college or above. Smoking status was defined as current, former, or never smoked within the past month; alcohol consumption status was defined as current, former, or never consumed within the past month. Moderate-to-vigorous physical activity duration: Participants were asked about daily moderate-to-vigorous physical activity duration over the past month. ≥ 1 hour/day was defined as sufficient physical activity, while <1 hour/day was defined as insufficient physical activity (20). Sleep duration: Participants were asked about nighttime sleep duration on rest days and school days during the past month. Sleep duration was calculated as (nighttime sleep duration on rest days ×2+ nighttime sleep duration on school days ×5)/7. Screen time: Participants were asked about daily screen time for electronic device use, television viewing, and video gaming during weekdays (Monday to Friday) and weekends in the past month. Daily screen time = (weekday daily screen time × 5 + weekend daily screen time × 2)/7. ≥ 2 hours/day was defined as excessive daily screen time, <2 hours/day as normal daily screen time (21). Carbonated beverage consumption: Participants were asked about carbonated beverage intake during the past month.

### Statistical analysis

Intergroup differences in baseline characteristics were compared using appropriate parametric or nonparametric tests according to variable type. Although variables such as smoking status, alcohol intake, ethnicity, and boarding at school were initially collected and considered as potential confounders, they were excluded from the final multivariable models. This decision was based on their highly skewed distributions and sparse event counts in this pediatric population (e.g., extremely low prevalence of current smoking and drinking). Including such sparse variables in logistic regression models could induce sparse-data bias and model instability. We employed a multivariable logistic regression model to assess the association between combined exposure to birth weight and current weight status with the clustering of CMRFs, reporting odds ratios (OR) and 95% confidence intervals (CI). Three regression models were constructed, progressively adjusting for potential confounders including sociodemographic and lifestyle factors.

To examine potential effect modification, subgroup stratification analyses and multiplicative interaction tests were conducted. Furthermore, the additive interaction between low birth weight and current overweight/obesity was assessed by calculating the relative excess risk ratio (RERI), attributable fraction (AP), and synergy index (SI). A statistical mediation model was further constructed, using current BMI as a potential mediating variable, to decompose the effect pathway of birth weight on the aggregation of CMRFs into direct and indirect statistical effects.

To validate the robustness of key findings, we conducted a series of sensitivity analyses: (1) Comparing baseline characteristics between the missing population and the final included population; (2) To account for potential selection bias arising from missing birth weight data without imputing the primary exposure variable, we performed an inverse probability weighting (IPW) analysis, in which the probability of having complete birth weight data was estimated using multivariable logistic regression and each included participant was weighted by the inverse of this probability before refitting the multivariable model; (3) We employed a Restricted Cubic Spline (RCS) model to examine potential nonlinear associations between continuous birth weight and outcomes.

All statistical analyses were performed using R software (version 4.3.0). Two-sided *P* < 0.05 was considered statistically significant.

## Results

### Baseline characteristics of participants

Of the 15,910 participants in the final analytical sample, the prevalence of LBW, NBW, and HBW was 2.23%, 82.53%, and 15.24%, respectively. Current overweight/obesity was present in 31.64% of participants. Overall, 6.30% of participants met the criteria for CMRF clustering. All participants were categorized into six combined exposure groups (Group 1-6) based on the combination of birth weight and current weight status. As shown in **Table 1**, the LBW/OB group (Group 4) comprised 89 participants (0.56% of total), representing the smallest exposure combination. In this specific group, 61.8% of participants resided in urban areas, and males accounted for 46.1%. Statistically significant differences existed across combined exposure groups for most sociodemographic and lifestyle variables, including age, sex, residence, primary caregiver education level, moderate-to-vigorous physical activity, screen time, and nighttime sleep duration (all *P* < 0.05, except for carbonated beverage consumption with *P* = 0.058).

**Table 1:** Baseline characteristics of participants by joint birth weight and current weight status categories.

	Group 1 (NBW/Non-OB)	Group 2 (LBW/Non-OB)	Group 3 (HBW/Non-OB)	Group 4 (LBW/OB)	Group 5 (NBW/OB)	Group 6 (HBW/OB)	*P value*
N, %	9157	266	1453	89	3973	972	
Age, y	11.86 (3.20)	12.20 (3.10)	12.24 (3.16)	12.37 (2.92)	11.80 (3.08)	11.76 (3.12)	<0.001
Sex, n (%)							
Male	4032 (44.0)	95 (35.7)	805 (55.4)	41 (46.1)	2280 (57.4)	638 (65.6)	<0.001
Female	5125 (56.0)	171 (64.3)	648 (44.6)	48 (53.9)	1693 (42.6)	334 (34.4)	
Residence, n (%)							
Urban	5794 (63.3)	162 (60.9)	861 (59.3)	55 (61.8)	2672 (67.3)	598 (61.5)	<0.001
Rural	3363 (36.7)	104 (39.1)	592 (40.7)	34 (38.2)	1301 (32.7)	374 (38.5)	
Ethnicity, n (%)							
Han	9017 (98.5)	261 (98.1)	1431 (98.5)	86 (96.6)	3905 (98.3)	958 (98.6)	0.740
Other	140 (1.5)	5 (1.9)	22 (1.5)	3 (3.4)	68 (1.7)	14 (1.4)	
Boarding at school, n (%)							
Yes	2808 (30.7)	94 (35.3)	494 (34.0)	27 (30.3)	999 (25.1)	253 (26.0)	<0.001
No	6349 (69.3)	172 (64.7)	959 (66.0)	62 (69.7)	2974 (74.9)	719 (74.0)	
Primary caregiver, n (%)							
Parents	8152 (89.0)	237 (89.1)	1289 (88.7)	80 (89.9)	3563 (89.7)	863 (88.8)	0.879
Other	1005 (11.0)	29 (10.9)	164 (11.3)	9 (10.1)	410 (10.3)	109 (11.2)	
Primary caregiver’s education, n (%)							
Primary school or below	1335 (14.6)	41 (15.4)	228 (15.7)	13 (14.6)	467 (11.8)	134 (13.8)	<0.001
High school	6082 (66.4)	179 (67.3)	987 (67.9)	66 (74.2)	2547 (64.1)	610 (62.8)	
College or above	1740 (19.0)	46 (17.3)	238 (16.4)	10 (11.2)	959 (24.1)	228 (23.5)	
Moderate-to-vigorous physical activity, n (%)							
Sufficient	7841 (85.6)	213 (80.1)	1248 (85.9)	74 (83.1)	3365 (84.7)	855 (88.0)	0.017
Insufficient	1316 (14.4)	53 (19.9)	205 (14.1)	15 (16.9)	608 (15.3)	117 (12.0)	
Screen time, n (%)							
Normal	5597 (61.1)	171 (64.3)	872 (60.0)	52 (58.4)	2333 (58.7)	554 (57.0)	0.023
Excessive	3560 (38.9)	95 (35.7)	581 (40.0)	37 (41.6)	1640 (41.3)	418 (43.0)	
Sleep duration, h	9.06 (1.20)	8.97 (1.33)	8.95 (1.26)	8.77 (1.14)	9.05 (1.22)	9.01 (1.28)	0.008
Carbonated beverage consumption, n (%)							
Yes	7912 (86.4)	233 (87.6)	1238 (85.2)	75 (84.3)	3363 (84.6)	817 (84.1)	0.058
No	1245 (13.6)	33 (12.4)	215 (14.8)	14 (15.7)	610 (15.4)	155 (15.9)	
Birth weight, g	3249.75 (321.86)	2092.86 (324.92)	4194.49 (312.60)	2060.67 (354.71)	3306.09 (324.16)	4185.29 (288.57)	<0.001
BMI, kg/m^2^	17.48 (2.44)	17.59 (2.56)	17.93 (2.38)	24.28 (3.72)	23.82 (4.09)	24.13 (4.14)	<0.001
WC, cm	59.45 (7.46)	59.65 (7.41)	60.99 (7.38)	75.71 (10.35)	74.93 (11.89)	76.24 (12.21)	<0.001
SBP, mmHg	107.33 (11.54)	108.32 (11.32)	108.51 (11.70)	115.37 (13.27)	115.46 (12.68)	115.33 (12.54)	<0.001
DBP, mmHg	65.81 (7.99)	66.15 (7.42)	65.80 (7.87)	68.57 (8.91)	68.34 (8.18)	67.87 (7.96)	<0.001
FBG, mmol/L	5.08 (0.47)	5.10 (0.41)	5.11 (0.43)	5.27 (1.19)	5.16 (0.50)	5.15 (0.46)	<0.001
TG, mmol/L	0.78 (0.34)	0.81 (0.36)	0.77 (0.33)	1.07 (0.57)	1.02 (0.53)	0.98 (0.52)	<0.001
HDL-C, mmol/L	1.66 (0.33)	1.63 (0.32)	1.65 (0.34)	1.37 (0.25)	1.45 (0.32)	1.46 (0.32)	<0.001
Clustered CMRFs, n (%)							
No	8865 (96.8)	257 (96.6)	1396 (96.1)	74 (83.1)	3451 (86.9)	865 (89.0)	<0.001
Yes	292 (3.2)	9 (3.4)	57 (3.9)	15 (16.9)	522 (13.1)	107 (11.0)	
Smoking status, n (%)							
Current	71 (0.8)	1 (0.4)	15 (1.0)	2 (2.2)	34 (0.9)	9 (0.9)	0.080
Ever	131 (1.4)	2 (0.8)	32 (2.2)	3 (3.4)	67 (1.7)	24 (2.5)	
Never	8955 (97.8)	263 (98.9)	1406 (96.8)	84 (94.4)	3872 (97.5)	939 (96.6)	
Alcohol intake, n (%)							
Current	286 (3.1)	8 (3.0)	68 (4.7)	1 (1.1)	136 (3.4)	54 (5.6)	<0.001
Ever	932 (10.2)	31 (11.7)	164 (11.3)	10 (11.2)	448 (11.3)	134 (13.8)	
Never	7939 (86.7)	227 (85.3)	1221 (84.0)	78 (87.6)	3389 (85.3)	784 (80.7)	

NBW, normal birth weight; LBW, low birth weight; HBW, high birth weight; OB, overweight/obese; Non-OB, non-overweight/obese.

Regarding physical and metabolic indicators, statistically significant differences were observed across groups for BMI, waist circumference, and biomarkers including fasting blood glucose (FBG), triglycerides (TG), and high-density lipoprotein cholesterol (HDL-C) (all *P* < 0.001). Furthermore, the prevalence of CMRF clustering showed significant differences across groups (*P* < 0.001), with the highest clustering rates observed in Group 4 (16.9%), followed by Group 5 (13.1%) and Group 6 (11.0%).

### Joint association with clustered CMRFs

**Table 2** presents the associations of joint birth weight–current weight status categories with CMRF clustering across three progressively adjusted models. In the fully adjusted model (Model 3), compared with the reference group 1 (NBW/Non-OB), significant positive associations with CMRF clustering were observed for group 5 (NBW/OB) (OR = 4.52, 95% CI: 3.89 to 5.26, *P* < 0.001), group 6 (HBW/OB) (OR = 3.57, 95% CI: 2.81 to 4.50, *P* < 0.001), and group 4 (LBW/OB) (OR = 6.11, 95% CI: 3.32 to 10.53, *P* < 0.001), with the last group exhibiting the highest point estimate. Among non-overweight groups, group 2 (LBW/Non-OB) showed a modestly elevated but non-significant association (OR = 1.06, 95% CI: 0.50 to 1.97, *P* = 0.865), whereas group 3 (HBW/Non-OB) was not associated with CMRF clustering (OR = 1.14, 95% CI: 0.84 to 1.51, *P* = 0.377). The ORs were slightly attenuated from Model 1 to Model 3, suggesting moderate confounding by sociodemographic and lifestyle factors.

**Table 2:** Associations of joint birth weight and current weight status with CMRF clustering.

Characteristic	Model 1		Model 2		Model 3	
	OR (95% CI)	*P*	OR (95% CI)	*P*	OR (95% CI)	*P*
Group 1	1.00 (Ref)	–	1.00 (Ref)	–	1.00 (Ref)	–
Group 2	1.06 (0.50–1.97)	0.859	1.06 (0.50–1.97)	0.861	1.06 (0.50–1.97)	0.865
Group 3	1.24 (0.92–1.64)	0.146	1.14 (0.84–1.51)	0.388	1.14 (0.84–1.51)	0.377
Group 4	6.15 (3.36–10.54)	<0.001	5.96 (3.24–10.26)	<0.001	6.11 (3.32–10.53)	<0.001
Group 5	4.59 (3.96–5.33)	<0.001	4.50 (3.87–5.23)	<0.001	4.52 (3.89–5.26)	<0.001
Group 6	3.76 (2.97–4.72)	<0.001	3.50 (2.76–4.42)	<0.001	3.57 (2.81–4.50)	<0.001

Model 1: unadjusted.

Model 2: adjusted for age, sex, primary caregiver’s education, and residence.

Model 3: additionally adjusted for moderate-to-vigorous physical activity, screen time, sleep duration, and carbonated beverage consumption.

### Subgroup analysis

**Figure 2:**
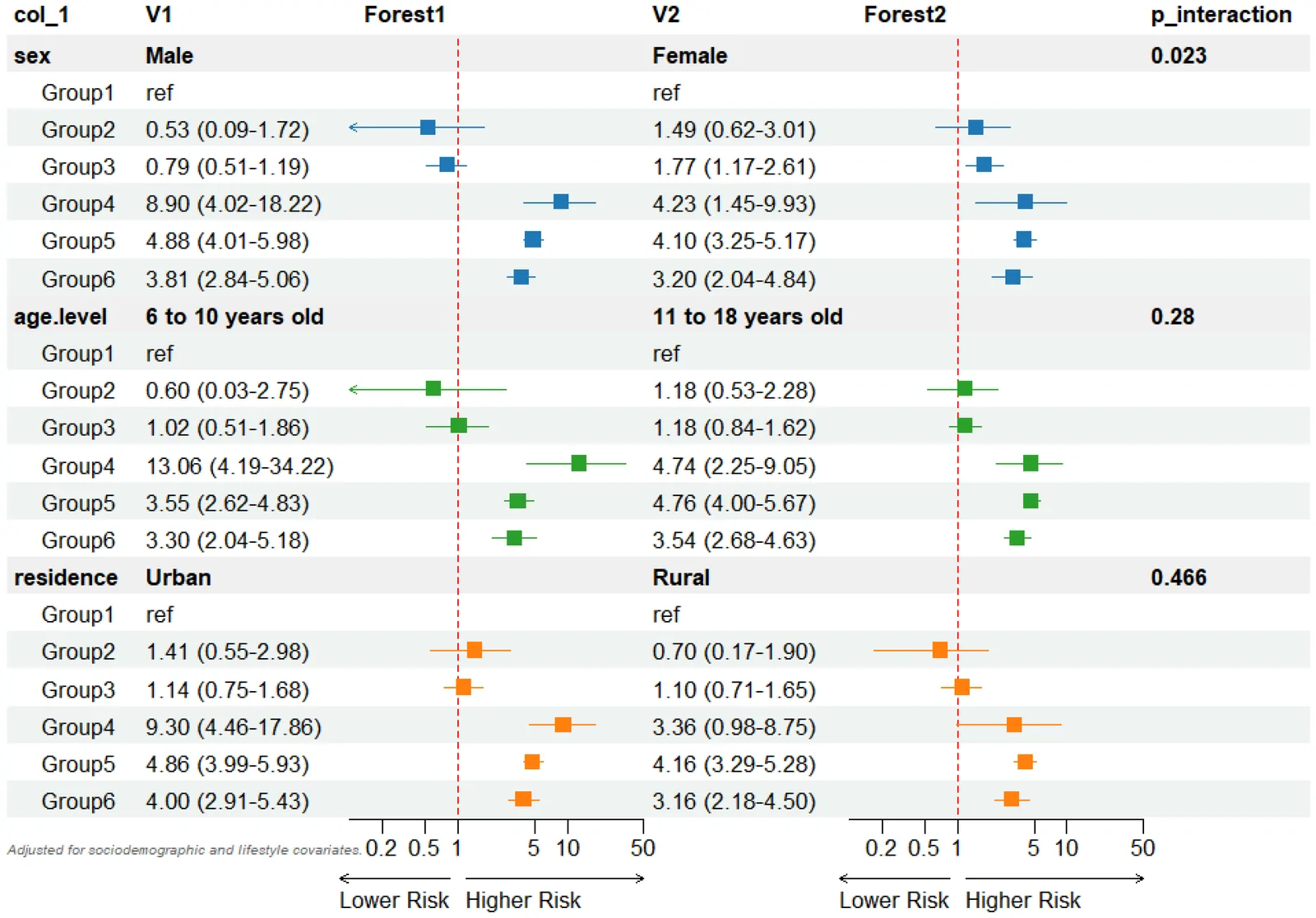
Subgroup analysis of the combined effect of birth weight and current overweight/obesity on risk of CMRFs. Models were adjusted for residence, sex, age, primary caregiver’s education level, moderate-to-vigorous physical activity, screen time, sleep duration, and carbonated beverage consumption.

To assess the potential modifying effects of sex, age, and residence on the aforementioned association, we conducted stratified analyses (**Figure 2**). Results indicated that sex significantly modified the association between combined exposure and the risk of CMRF clustering (interaction *P* = 0.023). Specifically, compared with the reference group, the pooled risk of CMRFs in Group 4 was substantially higher among males (OR = 8.90, 95% CI: 4.02 to 18.22) than among females (OR = 4.23, 95% CI: 1.45 to 9.93). Interaction tests for age group (interaction *P* = 0.280) and urban/rural residence (interaction *P* = 0.466) were not statistically significant, indicating that this association was relatively consistent across these strata. However, numerically, the point estimate risk for Group 4 status was more extreme in the younger age group (6–10 years; OR = 13.06, 95% CI: 4.19 to 34.22) compared to the older age group (11–18 years; OR = 4.74, 95% CI: 2.25 to 9.05).

### Additive interaction and mediation analysis

To further elucidate the pathways underlying the extremely high risk in Group 4 (low birth weight combined with acquired obesity), this study conducted an in-depth assessment of additive interactions and statistical mediating effects (**Table 3**). Additive interaction analysis revealed no statistically significant interaction between low birth weight and current overweight/obesity on CMRF clustering: RERI = 1.57 (95% CI: −1.97 to 5.11, *P* = 0.193), AP = 0.26 (95% CI: −0.18 to 0.70, *P* = 0.127), and SI = 1.44 (95% CI: 0.71 to 2.94, *P* = 0.158). Although the point estimates for all three indices were in the direction consistent with positive additive interaction (RERI > 0, AP > 0, SI > 1), the wide confidence intervals preclude definitive conclusions, likely due to the limited sample size in the LBW/OB group (n = 89). This indicates that the elevated risk in this subgroup primarily results from the physical summation of the strong independent effect of current obesity and the weak independent effect of low birth weight, with current obesity exerting absolute dominance. Mediation analysis (**Table 3**, Panel B) revealed a significant competitive mediation pattern between birth weight and CMRF clustering. With current continuous BMI as the mediating variable, birth weight exhibited a significant positive statistical indirect effect (*β* = 0.0091, *P* < 0.001), indicating that increased birth weight correlates with higher current BMI, which in turn correlates with increased metabolic risk. However, after controlling for current BMI, birth weight itself exhibited a significant negative statistical direct effect on CMRF clustering (*β* = −0.0144, *P* = 0.002), suggesting that lower birth weight independently contributes to metabolic risk predisposition.

**Table 3:** Additive interaction and mediation analysis.

Analysis/measures	Estimate/*β*	95% CI	*P*
Panel A: Additive interaction			
RERI	1.57	-1.97, 5.11	0.193
AP	0.26	-0.18, 0.70	0.127
SI	1.44	0.71, 2.94	0.158
Panel B: Mediation analysis			
Indirect effect	0.0091	0.0062, 0.0124	<0.001
Direct effect	-0.0144	-0.0304, -0.0035	0.002
Total effect	-0.0052	-0.0186, 0.0031	0.326

RERI, Relative excess risk due to interaction; AP, Attributable proportion; SI, Synergy index.

### Sensitivity analyses

To validate the robustness of our findings, we conducted a series of rigorous sensitivity analyses (see **Supplementary Materials** for details). First, we compared baseline characteristics between the 11,078 individuals excluded due to missing birth weight data and the 15,910 individuals ultimately included in the analysis (**Supplementary Table 1**). Results indicate that while differences in sex and current BMI were small (SMD ≈ 0.1), significant systematic imbalances existed in age (SMD = 0.380) and primary caregiver education level (SMD = 0.425). The proportion of primary caregivers with an education level of elementary school or below was significantly higher in the exclusion group (27.1%) than in the inclusion group (13.9%). Considering this substantial selection bias, we performed an inverse probability weighting (IPW) analysis to account for the probability of having complete birth weight data (**Supplementary Table 2**). The IPW analysis produced results consistent with the primary analysis: the low birth weight/overweight-obese group maintained the highest risk (OR = 5.72, 95% CI: 3.61-8.72), followed by the normal birth weight/overweight-obese (OR = 4.57, 95% CI: 4.07-5.12) and high birth weight/overweight-obese (OR = 3.57, 95% CI: 2.98-4.25) groups, indicating that the observed associations were not materially affected by selection bias. Furthermore, RCS analysis of birth weight as a continuous variable showed no significant overall or nonlinear association with the risk of CMRF clustering (**Supplementary Figure 1**).

## Discussion

This study, based on representative cross-sectional data from 15,910 children and adolescents in Henan Province, China, indicates that low birth weight combined with current overweight or obesity (Group 4) was associated with the highest odds of CMRF clustering. Additive interaction and mediation models reveal that this extreme risk does not stem from biological synergistic amplification between intrauterine growth restriction and current obesity, but is dominated by the strong independent effect of current obesity. After controlling for the mediating pathway of current BMI, low birth weight itself still independently exhibits a negative statistical direct effect increasing metabolic risk.

Our findings align strongly with the DOHaD hypothesis (6). Existing epidemiological and clinical evidence indicates that individuals with low birth weight are highly susceptible to catch-up growth in early life (22, 23). This rapid compensatory weight gain is often accompanied by ectopic accumulation of adipose tissue throughout the body—particularly visceral fat—which in turn induces underlying insulin resistance (24) and endothelial remodeling (25, 26). The extreme risk point estimate observed in Group 4 of this study (fully adjusted model OR = 6.11) provides a direct quantitative reflection of this “intrauterine undernutrition-postnatal overnutrition” developmental mismatch pattern at the epidemiological level.

Further additive interaction analysis ruled out any statistical synergistic amplification effect between the two factors, suggesting that current overweight and obesity may play a predominant role in the progression of metabolic risk. These findings suggest potential public health relevance: because intrauterine growth restriction represents an irreversible historical exposure, preventing excessive weight gain in children born with low birth weight—who are prone to catch-up growth—may be an important strategy for reducing their cardiometabolic risk. Simultaneously, mediation analysis indicates that even when fully controlling for current BMI gain, low birth weight itself retains metabolic harm. Biologically, this may stem from substantial organ developmental remodeling caused by intrauterine growth restriction—such as permanent reduction in pancreatic *β*-cell mass (27), impaired nephron generation (28), and abnormal sympathetic nervous system activation (29, 30). These embryonic structural fine-tuning processes establish a foundational high susceptibility to cardiovascular disease throughout the individual’s entire lifespan (31).

This study has certain limitations. First, the cross-sectional design precludes establishing strict causal relationships, although our mediation model effectively quantified statistical pathways; future longitudinal studies are warranted to validate these findings. Second, birth weight was retrospectively reported by the primary caregiver, which may introduce both missing data and recall bias. While our inverse probability weighting sensitivity analysis confirmed the high stability of our results, potential selection bias cannot be completely ruled out. To minimize recall bias, caregivers were instructed to refer to the child’s birth medical certificate, although some degree of misclassification may remain. Finally, sample sparsity in specific exposure combinations (e.g., low birth weight combined with obesity) led to wide confidence intervals in certain subgroups, requiring cautious interpretation and confirmation in larger cohorts.

## Conclusions

In this large cross-sectional study of Chinese children and adolescents, the combination of low birth weight and current overweight/obesity was associated with the highest risk of CMRF clustering, though this appeared to reflect the accumulation of independent effects rather than biological synergy. Current overweight/obesity was the predominant contributor to CMRF clustering risk. Importantly, low birth weight demonstrated an independent direct association with metabolic risk after accounting for current adiposity, supporting the DOHaD hypothesis and highlighting the need for enhanced cardiometabolic surveillance in this subpopulation. Preventing excessive weight gain in children born with low birth weight represents a potentially high-yield preventive strategy. Prospective longitudinal studies with serial growth measurements are warranted to confirm these findings and elucidate the underlying causal pathways.

## Data Availability

The raw data supporting the conclusions of this article will be made available by the authors, without undue reservation.
