# Supplementary material for: Combined association of birth weight and current overweight/obesity with cardiometabolic risk factor clustering in Chinese children and adolescents: a cross-sectional study
Source: Front Endocrinol (Lausanne). 2026 Sep 9;17:1917806. doi: 10.3389/fendo.2026.1917806 (PMC13597339; doi:10.3389/fendo.2026.1917806)
Supplement: Supplementary file 1 [file Supplementaryfile1.docx]

**Combined Association of Birth Weight and Current Overweight/Obesity with Cardiometabolic Risk Factor Clustering in Chinese Children and Adolescents: A Cross-Sectional Study**

Gang Tian ^#a^, Ruiheng Zhang ^#b^, Jialin Sun ^#c^, Xuejiao Chen ^#a^, Xiaozhuan Liu ^a^, Yajing Guo ^b^, Mengwei Wang^d^, Li Gao^d^, Chen Zhang ^c^, Huiyu Du^a f^, Zhicheng Han ^a f^, Jialiang Zhu ^e^, Dandan Tian^c^, Jingge Zhao^g^, Linqi Diao*^d^, Min Liu *^c^, Yibin Hao *^a b^

^a^ Henan Province Hypertension Precision Prevention and Control Engineering Research Center, Henan Provincial People's Hospital, Zhengzhou University People's Hospital, Zhengzhou, Henan, China.

^b^ College of Public Health, Zhengzhou University, Zhengzhou, Henan, China.

^c^ Department of Hypertension, Henan Provincial People's Hospital, Zhengzhou University People's Hospital, Zhengzhou, China.

^d^ Henan Provincial Center for Disease Control and Prevention, Zhengzhou, China.

^e^ Department of Cardiology, Henan Provincial People's Hospital, Zhengzhou University People's Hospital, Zhengzhou, Henan, China.

^f^ Henan Engineering Technology Research Center for Prevention and Treatment of Panvascular Diseases, Henan Provincial People’s Hospital, Zhengzhou University People’s Hospital, Zhengzhou, Henan, China.

^g^ Department of Clinical Research Center, Henan Provincial People's Hospital, Zhengzhou University People's Hospital, Zhengzhou, Henan, China.

**Short Title:** Birth Weight, Obesity, and CMRF Clustering

**Corresponding Author:**

**Yibin Hao*：**E-mail address: haoyibin0708@163.com

**Min Liu*：**E-mail address: liumin136@126.com

**Linqi Diao*:** E-mail address: lqdiao @163.com

**Keywords:** Birth weight; Childhood obesity; Cardiometabolic risk factor clustering; Interaction; Cross-sectional study

**Supplementary Material**

**Supplementary Table 1.** Baseline characteristics comparison between included participants and those excluded due to missing birth weight.

| **Characteristics** | **Excluded (Missing BW)(n = 11,078)** | **Included (Final Sample)(n = 15,910)** | ***P* Value** | **SMD** |
| --- | --- | --- | --- | --- |
| **Age (years), median [IQR]** | 13.56 [10.45, 15.78] | 11.63 [9.19, 14.48] | <0.001 | 0.38 |
| Females, No. (%) | 5,057 (45.6) | 8,019 (50.4) | <0.001 | 0.095 |
| Rural residence, No. (%) | 4,773 (43.1) | 5,768 (36.3) | <0.001 | 0.14 |
| **Primary caregiver's education, No. (%)** | |  | <0.001 | 0.425 |
| Primary school or below | 2,845 (27.1) | 2,218 (13.9) |  |  |
| High school | 6,707 (63.9) | 10,471 (65.8) |  |  |
| College or above | 937 (8.9) | 3,221 (20.2) |  |  |
| Missing | 589 |  |  |  |
| **BMI (kg/m2), median [IQR]** | 19.36 [16.95, 22.12] | 18.76 [16.34, 21.83] | <0.001 | 0.105 |

Notes: Continuous variables with skewed distribution are presented as median [interquartile range, IQR] and were compared using the Wilcoxon rank-sum test. Categorical variables are presented as counts (percentages) and were compared using the Pearson's Chi-square test.

Abbreviations: BW, birth weight; IQR, interquartile range; BMI, body mass index; SMD, standardized mean difference.

Percentages for primary caregiver's education level were calculated among participants with non-missing data; education data were missing for 589 participants in the excluded group.

**Supplementary Table 2.** Joint association of birth weight and current weight status with clustered cardiometabolic risk factors in the inverse probability weighting (IPW) analysis (n = 15,910).

| **Joint Exposure Group** | **IPW OR (95% CI)** | ***P*** |
| --- | --- | --- |
| **Normal / Non-overweight (Ref)** | **1.00** | **–** |
| **Low / Non-overweight** | **1.02 (0.58-1.65)** | **0.946** |
| **High / Non-overweight** | **1.07 (0.85-1.32)** | **0.562** |
| **Low / Overweight or obese** | **5.72 (3.61-8.72)** | **<0.001** |
| **Normal / Overweight or obese** | **4.57 (4.07-5.12)** | **<0.001** |
| **High / Overweight or obese** | **3.57 (2.98-4.25)** | **<0.001** |

Notes: To account for potential selection bias arising from missing birth weight data, the probability of having complete birth weight data was estimated using multivariable logistic regression, and each included participant was weighted by the inverse of this probability. Models were fully adjusted for residence, sex, age, primary caregiver's education level, moderate-to-vigorous physical activity, screen time, sleep duration, and carbonated beverage consumption.

Abbreviations: OR, odds ratio; CI, confidence interval; BW, birth weight.

**Supplementary Figure 1**. Dose-response relationship between continuous birth weight and the risk of clustered cardiometabolic risk factors.

The solid red line represents the estimated odds ratios (ORs), and the shaded salmon area represents the 95% confidence intervals (CIs). The horizontal dashed line indicates the reference value (OR = 1.0). The vertical dashed line indicates the reference point of birth weight where the OR is 1.0. The light blue histogram at the bottom displays the frequency distribution of birth weight across the study population. The restricted cubic spline model was fitted with 4 knots and fully adjusted for residence, sex, age, primary caregiver's education level, moderate-to-vigorous physical activity, screen time, sleep duration, and carbonated beverage consumption.

Abbreviations: CMRF, cardiometabolic risk factor; OR, odds ratio; CI, confidence interval.
